# Physical therapy for muscle strengthening in individuals with amyotrophic lateral sclerosis: A protocol for a systematic review and meta-analysis

**DOI:** 10.1371/journal.pone.0307470

**Published:** 2024-07-22

**Authors:** Aline Alves de Souza, Stephano Tomaz da Silva, Lorenna Raquel Dantas de Macedo, Diogo Neres Aires, Karen de Medeiros Pondofe, Luciana Protásio de Melo, Ricardo Alexsandro de Medeiros Valentim, Tatiana Souza Ribeiro

**Affiliations:** 1 Department of Physical Therapy, Federal University of Rio Grande do Norte, Natal, RN, Brazil; 2 Laboratory of Technological Innovation in Health, Federal University of Rio Grande do Norte, Natal, RN, Brazil; 3 Department of Biomedical Engineering, Federal University of Rio Grande do Norte, Natal, RN, Brazil; Lorestan University, ISLAMIC REPUBLIC OF IRAN

## Abstract

**Introduction:**

People with Amyotrophic Lateral Sclerosis (ALS) can present initially muscle weakness, which is a debilitating symptom that may be improved by engaging in muscle strengthening activities. Currently, the effects of motor interventions for muscle strengthening in people with ALS are unclear. This review intends to analyze the effects of motor interventions for muscle strengthening in individuals with ALS

**Methods and analysis:**

Randomized, non-randomized, and quasi-experimental clinical trials assessing individuals with ALS of both sexes, aged 18 years or older, who have received motor interventions for muscle strengthening considering all practices that can lead to increased strength, endurance, power and muscular hypertrophy will be included. No restriction on language, location, or publication date will be applied. MEDLINE, EMBASE, Cochrane Library (CENTRAL), SPORTDiscus, and Physiotherapy Evidence Database (PEDro) databases will be searched. The US National Institutes of Health Ongoing, ClinicalTrials.gov, and the reference lists of included studies will also be searched. Two reviewers will independently screen titles and abstracts and extract data from included studies. The methodological quality of the included studies will be assessed by the PEDro scale and the certainty of the evidence by the GRADE approach. Disagreements will be resolved by a third researcher. Findings will be presented in text and table formats. A meta-analysis will compare the effects of motor interventions for muscle strengthening versus placebo or other interventions.

## Introduction

Amyotrophic lateral sclerosis (ALS) is a progressive neurodegenerative disease characterized by impaired upper and lower motor neurons of the cortex, brainstem, and spinal cord. ALS can be classified according to the onset of symptoms as spinal or bulbar [1, 2]. The global prevalence of ALS is four to six cases per 100,000 people, with the spinal type being more prevalent in 58% to 82% of cases [3].

Symptoms may vary according to the disease onset site that is an important driver of the disease progression [4]. Symptoms of spinal ALS appear initially in the upper or lower limbs in the form of muscle weakness, fatigue, or pain. Symptoms of bulbar ALS are related to the cerebral bulb and include facial muscle weakness, tongue and lip fasciculations, dysphagia, dysarthria, and respiratory muscle involvement, which may lead to death due to respiratory failure [5]. Respiratory complications, such as aspiration pneumonia, may also be related to weakness in the orofacial muscles of individuals with ALS [6]. The progressive onset of muscle weakness observed in spinal ALS reduces functional performance [7] and affects mobility, daily activities, and quality of life [8].

Healthy individuals may present disuse, i.e., muscle atrophy and weakness, with reduced physical activity levels, as a result of decreased activity/inactivity/reduced muscle demand for long periods [9]. In contrast, individuals with ALS experience neurological deterioration due to excessive demand of the muscles, known as overuse [10]. It is known that a debilitating symptom of ALS is peripheral muscle weakness, which may be improved by physical therapy undertaking muscle strengthening interventions, aiming to reduce impairments and improve function and quality of life and can be tailored to individual needs and abilities [11].

Therapeutic exercises when properly prescribed and considering the ALS stage promote physiological and psychological benefits in this population. Therefore, low to moderate-intensity aerobic and resistance exercises focused on muscle groups that are less affected or unaffected by the disease can be beneficial, especially in the early and middle stages of ALS [12]. Those exercises may preserve trunk and limb muscle strength and improve aerobic fitness and function.

The most recent evidence demonstrates that aerobic exercises can enhance the functional capacity of individuals with ALS. Additionally, therapeutic exercises can help reduce muscle deterioration in people with ALS and facilitate the performance of daily life activities [13]. A systematic review has shown that combined aerobic and resistance exercises positively affect muscle strength. However, the included studies considered all health improvement interventions without focusing solely on muscle strength [14]. It is worth noting that the majority of available evidence for this population focuses on exercise programs with a predominance of aerobic modalities, passive exercises, and stretching, with relatively limited attention given to muscle strengthening exercises [14, 15], which are interventions that can lead not only to strength improvement, but also increased endurance, power and muscular hypertrophy in individuals with ALS [16].

Recent studies have shown that the skeletal muscle plays an important role in ALS, having a direct impact on progressive muscle weakness, beyond what occurs only solely as a response to denervation [17]. Therefore, the development of treatment programs should consider the clinical phenotypes of ALS, individual characteristics, disuse and overuse expression, and the rapid, progressive, and deteriorating nature of the disease, mainly targeting muscle strength to improve muscle function [17].

The lack of high-quality evidence on muscle strengthening for individuals with ALS may negatively influence the quality of rehabilitation currently delivered for this population. Thus, this study aims to analyze the evidence regarding the effects of motor interventions for muscle strengthening in individuals with ALS.

## Materials and methods

This systematic review protocol is reported according to the Preferred Reporting Items for Systematic Reviews and Meta-Analyses (PRISMA-P) [18]. The study was registered in the Open Science Framework (OSF) at https://doi.org/10.17605/OSF.IO/ZFA7H.

### Eligibility criteria

Eligibility criteria were established according to the PICOTS strategy (Participants, Interventions, Comparisons, Outcomes, Time, and Study design). Randomised, non-randomized and quasi-experimental clinical trials performed with adult patients, all ages, of both genders and diagnosed with definite, probable, or possible ALS [19] will be included if provided full-text or sufficient information about motor interventions for peripheral muscle strengthening. Studies with motor interventions for respiratory muscle strengthening will be excluded.

#### Study design

Randomized, non-randomized, and quasi-experimental clinical trials assessing motor interventions focused on muscle strengthening of the upper limbs, lower limbs, face, neck, and trunk in individuals with ALS will be included. Cross-over studies will be included if data from the motor interventions (control and experimental) are presented separately.

#### Interventions and comparisons

Any treatment modality or intervention aiming at muscle strengthening of the upper limbs, lower limbs, face, neck, and trunk muscles, such as therapeutic exercises (using free weight, machine, elastic band, dumbbells, body weight, using pneumatic devices) and electrostimulation will be considered. It will be considered muscle strengthening all practices that can lead to increased strength, endurance, power and muscular hypertrophy. Studies that performed interventions focused on respiratory muscles will not be included.

For the control group, it will be consider:

- any other intervention not aiming at muscle strengthening (e.g., passive mobilization exercises, stretching, relaxation, massage therapy, music therapy, among others);
- minimal intervention, including education about maintaining activities of daily living, booklets, and energy conservation techniques, among others;
- placebo or no intervention.

### Outcome measures

- The primary outcome will be peripheral muscle strength assessed by the manual muscle test (MMT) [20], muscle strength grading scale [20], isokinetic [21], or hand-held dynamometer [22].
- Secondary outcomes will include muscle activation, assessed by electromyography (EMG); function, assessed by the Amyotrophic Lateral Sclerosis Functional Rating Scale ‐ revised (ALSFRS-r) [23]; fatigue, assessed by the Fatigue Severity Scale (FSS) [24] or Borg Rating of Perceived Exertion (RPE) scale [25]; and adverse events, such as pain, discomfort, fatigue, or others reported by the included studies.

### Timepoints of assessments

The outcome data extracted from the included studies will refer to:

- baseline assessment (before intervention);
- final assessment (immediately after the intervention; acute effects);
- follow-up assessments, up to three months (short-term) or after three months (long-term) of the intervention.

### Search strategy

MEDLINE, EMBASE, Cochrane Library (CENTRAL), SPORTDiscus, and Physiotherapy Evidence Database (PEDro) databases will be searched. In addition, the US National Institutes of Health Ongoing and ClinicalTrials.gov (www.clinicaltrials.gov/) will be searched for trial registration, and potential studies will be screened from the reference lists of included studies. No restriction on language, location, and publication date will be applied.

The search strategy for the MEDLINE database is presented in the S1 Appendix and will be adapted for other databases according to study type (i.e., clinical trials), population (i.e., individuals with ALS), and interventions (i.e., muscle strengthening).

#### Data screening and extraction

Study selection, duplicate removal, and data extraction will be conducted using the RayyanⓇ (Intelligent Systematic Review) software [26]. Two reviewers (AAS and DNA) will screen titles and abstracts independently and remove duplicates. Next, potentially eligible studies will be read in full text and selected according to the inclusion criteria. Disagreements will be resolved through discussion, and a third author (STS) will be consulted if necessary. The screening will be recorded in a PRISMA flowchart [18].

The same two reviewers will extract data from the included studies using a data extraction sheet prepared by the researchers containing the first author, publication year, study design, sample size, characteristics of the participants, outcome measures, intervention characteristics, and results. The authors of the included studies will be contacted for clarifications or in case of missing data.

#### Risk of bias assessment

Two authors (AAS and STS) will assess the risk of bias in the included studies using the PEDro scale [27]. This instrument consists of 11 criteria assessing the methodological quality of randomized controlled trials and determines the quality of the study based on a score ranging from 0 to 10. Higher scores mean better methodological quality; scores below five are considered low methodological quality. Any disagreements will be resolved by a third reviewer (LRDM).

### Data analysis and processing

Statistical analyses will be conducted using Review ManagerⓇ web version. For continuous outcomes, the mean difference will be selected when tools and units of measurement are the same. The standardized mean difference will be used when the measurement tools and units differ. The data will be reported by the effect size and 95% confidence intervals. For dichotomous outcomes (adverse events), the odds ratio will be used to measure treatment effects with 95% confidence intervals.

#### Heterogeneity

Heterogeneity will be determined by χ^2^ and I^2^ values. If P ≥ 0,1, I^2^ ≤ 50%, which indicates low heterogeneity, the random effects model will be used for meta-analysis. If P < 0,1, I^2^ > 50%, which indicates heterogeneity between studies, the source of heterogeneity will be explored using subgroup analyses [28].

#### Meta-analysis

Meta-analyses using the random effects model will be performed if studies are sufficiently similar (i.e., clinically and methodologically), allowing to be pooled for analysis using Review ManagerⓇ web version. Studies will be pooled in the meta-analyses to compare the effects of:

- Motor interventions targeting muscle strengthening versus placebo;
- Motor interventions targeting muscle strengthening versus no treatment;
- Motor interventions or others targeting muscle strengthening versus any other intervention not aiming at muscle strengthening;
- Motor interventions targeting muscle strengthening versus educational programs and orientation.

#### Missing data

Authors of the included studies will be contacted via email in case of any missing data, for additional information regarding statistical data, or other relevant information. A descriptive analysis of the primary studies will be conducted if the necessary data for meta-analyses are not obtained, after contacting the corresponding authors.

#### Subgroup analysis

If the number of included studies is sufficient, the following variables will be considered for subgroup analyses:

- Type of ALS: spinal or bulbar;
- Duration of disease: less or more than five years;
- Age: less or more than 60 years old;
- Type of treatment: strengthening performed manually or by instruments (shin guards, machines, elastic bands).
- Frequency of treatment: one or two times per week or more than two times per week.

#### Sensitivity analysis

Sensitivity analyses will be performed if any missing data are suspected of bias and to assess heterogeneity caused by outlying studies. In addition, sensitivity analyses excluding studies with low scores in the risk of bias assessment (< 5) may be performed [25].

#### Certainty of the evidence

The Grading of Recommendations, Assessment, Development, and Evaluation (GRADE) approach will be used to assess the certainty of the evidence. The GRADE assesses five domains: study limitations, inconsistency, imprecision, indirectness, and publication bias of the included studies [29]. Two authors (AAS and STS) will do this independently through the GRADEpro Guideline Development Tool. All decisions to downgrade the quality of evidence will be justified with footnotes and comments to facilitate understanding. The quality of evidence will be graded as high, moderate, low, or very low [29].

#### Ethics and dissemination

This study design does not require ethical approval as it does not involve humans. The results will be published in peer-reviewed scientific journal publications.

#### Patient and public involvement

No individuals are involved in this study protocol.

#### Risk of publication bias

The risk of publication bias will be assessed by using the visual exploration of funnel plots, if more than ten studies are included.

## Discussion

The prescription of muscle strengthening interventions for individuals with ALS is still controversial regarding safety and effects, mainly due to the denervation and muscle degeneration resulting from overuse. Therefore, analyzing the effects of muscle strengthening exercises to delay and minimize motor impairments in individuals with ALS is necessary. This systematic review will synthesize the existing evidence on muscle strengthening for individuals with ALS and incorporate new insights into the role of muscle strengthening in managing ALS. The evidence may be limited, and acknowledgment is made of the possibility of retrieving few studies due to the rarity and rapid progression of the disease [5, 30, 31]. The results of this systematic review will benefit the scientific community, healthcare professionals, and people with ALS. Furthermore, it will contribute to the knowledge about ALS treatment options and support clinical decisions related to muscle strengthening for this population, an aspect that has been relatively underexplored in research for this population.

### Strengths and limitations

No previous systematic review examined the effects of different modes and types of motor interventions on muscle strength in individuals with ALS. Thus, this study aims to analyze the effects of muscle strengthening exercises on peripheral muscle strength and function in individuals with ALS. Additionally, it’s one of the aims to critically assess whether muscle strengthening is safe and effective for treating this population.

The limitations of this review may be related to intervention heterogeneity for this population. In addition, because clinical trials with this population are limited and considering the rarity and rapid progression of the disease, few studies with non-representative samples are expected.

## Supporting information

S1 Appendix Search strategy for MEDLINE. (DOCX)

S1 Checklist PRISMA-P 2015 checklist. (DOC)
